# The Role of MicroRNAs in Liver Functioning: from Biogenesis to Therapeutic Approaches (Review)

**DOI:** 10.17691/stm2023.15.5.06

**Published:** 2023-10-30

**Authors:** D.S. Kozlov, S.A. Rodimova, D.S. Kuznetsova

**Affiliations:** Laboratory Assistant, Scientific Laboratory of Molecular Biotechnologies, I Research Institute of Experimental Oncology and Biomedical Technologies; Privolzhsky Research Medical University, 10/1 Minin and Pozharsky Square, Nizhny Novgorod, 603005, Russia; Student, Institute of Biology and Biomedicine; National Research Lobachevsky State University of Nizhny Novgorod, 23 Prospekt Gagarina, Nizhny Novgorod, 603022, Russia; Junior Researcher, Laboratory of Regenerative Medicine; Privolzhsky Research Medical University, 10/1 Minin and Pozharsky Square, Nizhny Novgorod, 603005, Russia; Junior Researcher, Scientific Laboratory of Molecular Biotechnologies, Research Institute of Experimental Oncology and Biomedical Technologies; Privolzhsky Research Medical University, 10/1 Minin and Pozharsky Square, Nizhny Novgorod, 603005, Russia; PhD, Head of the Scientific Laboratory of Molecular Biotechnologies, Research Institute of Experimental Oncology and Biomedical Technologies; Privolzhsky Research Medical University, 10/1 Minin and Pozharsky Square, Nizhny Novgorod, 603005, Russia; Head of the Research Laboratory for Molecular Genetic Researches, Institute of Clinical Medicine; National Research Lobachevsky State University of Nizhny Novgorod, 23 Prospekt Gagarina, Nizhny Novgorod, 603022, Russia

**Keywords:** microRNAs, liver homeostasis, microRNAs biogenesis, non-canonical microRNAs, microRNA-based therapy, microRNA-based diagnostics, microRNA delivery systems

## Abstract

Molecular diagnostics based on small non-coding RNA molecules (in particular microRNA) is a new direction in modern biomedicine and is considered a promising method for identification of a wide range of pathologies at an early stage, clinical phenotype assessment, as well as monitoring the course of the disease, evaluation of therapy efficacy and the risk of the disease recurrence. Currently, the role of microRNAs as the most important epigenetic regulator in cancer development has been proven within the studies of normal and pathogenic processes. However, currently, there are insignificant studies devoted to studying the role of microRNAs in functioning of other organs and tissues, as well as to development of possible therapeutic approaches based on microRNAs. A huge number of metabolic processes in the liver are controlled by microRNAs, which creates enormous potential for the use of microRNAs as a diagnostic marker and makes it a target for therapeutic intervention in metabolic, oncological, and even viral diseases of this organ.

This review examines various aspects of biological functions of microRNAs in different types of liver cells. Both canonical and non-canonical pathways of biogenesis, epigenetic regulation mediated by microRNAs, as well as the microRNAs role in intercellular communication and the course of viral diseases are shown. The potential of microRNAs as a diagnostic marker for various liver pathologies is described, as well as therapeutic approaches and medicines based on microRNAs, which are approved for clinical use and currently being developed.

## Introduction

Liver is one of the most important organs responsible for homeostasis control in mammals. It is involved in various physiological processes, including bile production, plasma proteins synthesis, nutrients absorption, detoxification, and vitamins storage. The major reactions of carbohydrate, protein and lipid metabolism proceed in the liver, and it is also considered the most important immunological organ, capable of activating the immune system in response to circulating antigens. In addition to the large number of cell types in the liver structure, one can see a high functional specialization of hepatocytes. The functional unit here is the liver lobule. Based on modern understanding of the lobule structure, hepatocytes are located in three areas, specified per their location along the porto-central axis, which governs their functional profile. Recent studies based on high-throughput single-cell RNA sequencing combined with spatial transcriptomics and proteomics confirmed their high areal heterogeneity. Furthermore, it was established that the areal specialization of hepatocytes is very flexible and dynamic, it varies in accordance with multiple physiological signals, which requires active regulation of metabolic gene expression.

MicroRNAs are short RNA oligonucleotides of 20-22 bases long, which are involved in the expression regulation of, by various estimates, 30 to 60% of human genes. The enormous regulatory potential of microRNAs is based on their specific features: one microRNA molecule can have from hundreds to thousands of potential target genes [1, 2]; microRNA biogenesis is one of the fastest among cellular transcripts: at least 40% of microRNAs mature within 5 min [3]. Therefore, microRNAs create a complex, highly dynamic, context-dependent network of interactions with target transcripts. Approximately 2654 mature microRNA sequences in the human genome were identified and reported in miRbase 22.1 (https://www.mirbase.org/).

Molecular diagnostics based on the analysis of the following panels: transcripts, proteins, and metabolites are considered a promising method to identify a widest range of pathologies at an early stage, assess the clinical phenotype, as well as monitor the course of the disease, evaluate therapy efficacy and the risk of the disease recurrence. Due to the high dynamics of the microRNAs pool in the body, as well as their stability in samples of biological fluids and tissues, interest in microRNAs as a basis for diagnostic test systems persists for a decade and a half. Currently, the contribution of various microRNAs in liver homeostasis, pathologies development, and regeneration stimulation is actively studied, and therapeutic strategies for treatment of a wide range of liver diseases are developed. However, when defining the microRNAs function or developing microRNA-based therapies, authors often consider their functioning as the one of negative regulators of translation, without taking into account a wide range of non-canonical effects of microRNAs. At present, there are only few large reviews of non-canonical functioning of microRNAs [4-7]. One of the currently unsettled problems of the microRNA-based therapy is related to off-target effects that occur due to nonspecific microRNAs delivery or off-target genes regulation. Thus, creation of microRNAs-based therapeutic strategies requires further studies of the microRNA-target interaction network in various liver cell populations, as well as examination of non-canonical functions of microRNAs in liver homeostasis.

This review tackles biogenesis of microRNAs, features of their encoding, transcription, and maturation, classical and non-classical mechanisms of their operation, as well as clinical approaches to treatment and diagnosis of liver diseases.

## Biogenesis of microRNAs

Reviews [8-11] describe in detail the current understanding of the microRNA biogenesis pathways. We shall briefly consider the main provisions thereof.

### Features of microRNA encoding

Genes encoding microRNAs can be divided into several categories depending on their location in the genome and, respectively, the properties related to encoding at a specific locus.

The majority of currently known human microRNAs described in the miRBase are encoded in intergenic regions (68%). Most intragenic microRNAs are intronic (12% of all genes). Other microRNA genes are located in repeats, long non-coding RNAs (lncRNAs), untranslated regions (UTRs), or encoding regions of host genes [12]. Non-canonical microRNAs can also rise from other non-coding RNAs (tRNAs, small nucleolar RNAs, etc.), mitochondrial microRNAs, and viral microRNAs [13, 14]. The role of these RNAs in the regulation of cell processes is still underexplored. As microRNA transcriptional activity greatly depends on the genomic context, we decided to consider several examples. The role of microRNAs encoded by the viral genome shall be discussed in a separate section.

MicroRNA genes are often located in the so-called clusters. Moreover, microRNAs can be encoded within the genes of other cell transcripts and can be co-transcribed, which is confirmed by the appropriate correlation of the expression patterns for several genes [15]. Here, intronic microRNAs can be transcribed independently of their host gene, whereas polycistronic microRNA transcripts can be subject to alternative splicing to obtain specific microRNA expression [16, 17]. MicroRNA-17-92, one of the most precisely characterized microRNA clusters, is well known for its important role in cell differentiation and ontogenesis of the mammals. Contrary to ideas on regulation of the microRNA clusters expression, members of this cluster can be expressed both in a coordinated and in a separate manner [18, 19]. Dysregulation of this cluster and its individual constituents in the liver may contribute to development of hepatocellular carcinoma (HCC) as well as to progression of various liver pathologies. Impaired expression of microRNA-17 can result in the steatosis development [20].

Co-regulated mRNA and intron-encoded microRNA can be exampled by microRNA-33. In humans, microRNA-33a and microRNA-33b are encoded in the genes’ introns, which in turn encode sterol regulatory element binding proteins (SREBP-2 and SREBP-1); in mice, only one isoform of microRNA-33 (located in the intron of SREBP-2) is expressed. SREBP transcription factors, like microRNA-33, are key regulators of lipid metabolism and transport [21, 22]. Members of the microRNA-33 family are the first identified microRNAs that regulate lipoprotein homeostasis [21,23].

MicroRNAs encoded within lncRNAs are also worth noting. An example of them is microRNA-155, which encodes the miPEP155 regulatory peptide [24]. Unlike plant cells, where similar peptides regulate microRNAs expression with which they were encoded, miPEP155 does not affect the level of microRNA-155, rather it modulates antigen presentation by activated antigen-presenting cells through interaction with the chaperone protein HSC70, which affects its antigen transporter function in dendritic cells [25].

### MicroRNAs transcription and maturation

**Figure 1.**
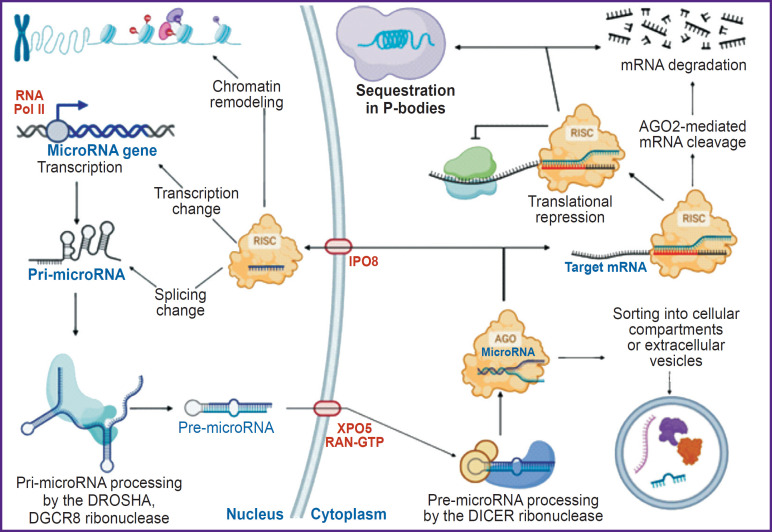
Canonical biogenesis and variants of microRNA functioning

MicroRNA maturation is traditionally divided into three stages: a long precursor (pri-microRNA), which, after splicing and processing, is sent to the cytoplasm in the form of a hairpin (pre-microRNA), giving rise to a mature microRNA. The main stages of microRNA biogenesis and functioning are demonstrated in Figure 1.

Canonical microRNAs in animals are transcribed by RNA polymerase II as long primary microRNA transcripts (pri-microRNAs). Hairpin structures in pri-microRNAs are cleaved by the DROSHA and DGCR8 endonuclease complexes thus forming hairpin pre-microRNAs. Transport of pre-microRNA from the nucleus to the cytoplasm is mediated by exportin 5 (XPO5) and RAN-GTP, although other export mechanisms are also suggested [26]. Pre-microRNA enters the cytoplasm and is cleaved near the terminal loop by RNase III DICER according to cleavage rules that consider the distance from the end of the hairpin fixed in the PAZ domain, as well as various mismatches in the hairpin stem [27, 28]. Moreover, recent large-scale analytic studies of pre-microRNA variants identified a conserved GYM motif (where G is paired guanine, Y is paired pyrimidine, M is unpaired cytosine, or adenine) adjacent to the human DICER cleavage site. The GYM motif is recognized by the dsRNA binding domain (dsRBD) of DICER and can ignore other cleavage rules [29].

The microRNA duplex is loaded into Argonaute (AGO) proteins (AGO1-AGO4 in mammals). The AGO1 and AGO2 proteins are the most expressed both in the liver and in other tissues [30, 31]. Once loaded, only one strand, called the guide strand, whose 5’ nucleotide interacts with the MID domain of the AGO proteins, is kept for further formation of a final complex called the RNA-induced silencing complex (RISC) [32].

The origin of the microRNA strand from different hairpin arms determines the name of the microRNA mature form. The 5p strand results from 5’ end of the pre-microRNA hairpin, whereas the 3p strand — from 3’ end. Both strands derived from a mature microRNA duplex can be loaded into the Argonaute proteins family in an ATP-dependent manner. For any microRNA, the proportion of AGO-loaded 5p or 3p strands significantly varies depending on the cell type or cellular environment, ranging from almost equal proportions to a predominance of one of them [33].

In addition to different lengths due to various scenarios of pre-microRNA processing by DICER, the repertoire of microRNA isoforms is enriched by introduction of terminal modifications and A-to-I (adenine to inosine) bases editing [34]. Such events are often perceived as artifacts of high-throughput sequencing [35], but many iso-microRNAs are found in cells in a proportion equal to the level of canonical microRNA or even higher than this level, which raises the question of their biological significance and role in pathologies development. It was demonstrated [36] that iso-microRNAs are functional in a cell, and isoforms with 5’ modifications have an altered repertoire of targets due to a shift in the target recognition region compared to 3’ isoforms, which can also lead to changes in the list of regulated transcripts [37] and influence their affinity for the target. For instance, Park et al. [38] found that the 5’ isoform of microRNA-21-5p is clinically significant in the HCC development and progression due to suppression of the growth hormone receptor (GHR) expression. Biogenesis of microRNA isoforms is described in details in the review [39].

Identification of mechanisms of microRNA processing and generation of microRNA isoforms is a key priority for understanding their biological function, as well as creating analytic systems and therapeutic strategies [40, 41].

## Mechanism of microRNA functioning

### Translational repression mediated by microRNAs

Suppression of protein synthesis on the mRNA template is translational repression mediated by microRNAs, which can occur through several independent pathways, the final result of which is destruction of the structure that brings together 3’ and 5’ ends of the mRNA. Both pathways can act both independently or jointly.

The guide strand and the AGO protein form the minimal RISC effector complex (miRISC). MicroRNA directs miRISC to specifically recognize mRNA and post-transcriptionally regulate gene expression. Recognition is conducted using the microRNA seed region. Bases 2-7 or 2-8 of microRNA interact through Watson-Crick base pairing with complementary sequences of the target mRNA called microRNA response elements (MREs). Mismatches in the seed region and its low affinity may require additional complementarity sites for effective silencing [42]. The majority of microRNA binding sites are located in the 3’ untranslated region (UTR) of the target mRNA. However, microRNA binding sites were also found in 5’UTR sequences, in encoding and promoter regions. In addition to the classical sites at positions 2-8 of microRNA bases, there are alternative mechanisms for target recognition. For microRNA-122, there were 1923 target transcripts identified by a non-classical binding site with a G-bulge formation. This gives 18.7% of transcripts regulated by the ten most expressed microRNAs in the liver [43].

The degree of microRNA complementarity to the target determines the mechanism of gene silencing. MicroRNA-directed cleavage of mRNA induced by high sequence complementarity is catalyzed by AGO2. Only a relatively small part (<6%) of all microRNAs in mammalian cells are susceptible to this mechanism [44]. At that, nucleotide mismatches in the central region and positions 17-21 during base pairing prevent AGO2 endonuclease activity but initiate recruitment of proteins that promote mRNA decay by means of deadenylating, decapping, and exonucleolytic cleavage [45-47]. The detailed mechanism of translational repression variants is provided in the review [48]. The main aspects are provided below.

The miRISC effector complex can inhibit translation initiation in several sequential stages. In most cases, the first stage is the reversible release of 4A eukaryotic initiation factors (eIF4aI and eIF4aII) from the mRNA-protein complex, which prevents the assembly of the eIF4F translation initiation complex [49], as well as recognition of the translation initiation site. This mechanism of microRNA-mediated translational repression is a sequestration of mRNA from the translation machinery into cytoplasmic processing bodies (P-bodies), which are the functional site of microRNA-mediated gene silencing. P-bodies have no translation mechanism and, therefore, do not participate in the translation process; however, they ensure sequestration and fast inclusion of important regulatory proteins and translation factors into the actively transcribed pool of mRNA [50, 51].

Then, one sees irreversible degradation with removal of the 3’-poly(A) tail (deadenylating) and 5’-cap structure (decapping) of the mRNA, which makes it open to exo- and endonucleases. TNRC6 (GW182) proteins, partners of AGO, play an important role in target repression by interacting with poly(A) binding protein and bringing deadenylation complexes PAN2-PAN3 and CCR4- NOT to target mRNAs [52, 53]. Decapping exposes the 5’ end of the mRNA to degradation by the conservative 5’→3’ cytoplasmic exonuclease XRN1, recruited to the target mRNA through direct interaction with decapping protein 1 (DCP1), which ensures fast removal of decapped mRNAs [54]. Shortening or removal of the poly(A) tail of mRNA can be a signal for decapping, as it promotes recruitment of the Lsm1-Lsm7 and PatL1 proteins, which activate the decapping complex assembly [55].

Currently, interaction of several microRNAs with one target, which can mediate competition between microRNAs, is actively studied [56, 57]. It was demonstrated [58, 59] that microRNA binding sites separated by a maximum of 26 nucleotides can act cooperatively. The human transcriptome is enriched with microRNA binding sites located at a distance that allows cooperativity. Surprisingly, some microRNAs are destabilized by specific interactions with mRNA [60-62]. These transcripts contain sequences that almost completely match microRNAs and, in turn, contain central mismatches. This type of interaction makes microRNA unload from the AGO and destabilizes the 3’ end of the microRNA. This post-transcriptional regulation of microRNAs is also called target-directed microRNA degradation (TDMD). In contrast to cleavage induced by the catalytic activity of AGO2, TDMD requires complete 3’ complementarity, which opens microRNAs to enzymes [63].

To effectively predict microRNA targets and design artificial and small interfering (siRNA) microRNAs that target single transcripts as the result of the extended area of complementarity, it is necessary to understand thermodynamic stability of the microRNA-target duplex, as well as the structure of protein domains that are included in the RISC complex [64]. The range of microRNA targets, as well as the mechanism of translational repression, shall be determined by the composition of the RISC complex and the interaction between the pool of targets and microRNAs in the cell [65]. Reviews [66, 67] contain descriptions of the most recent algorithms to predict microRNA targets and the most popular resources based on both the *in silico* analysis and experimental data, but each experiment must include stages to control off-target effects and efficacy of target transcript silencing.

### Non-classical mechanisms of microRNA functioning

Studies of the last 10 years demonstrate the significant role of non-canonical mechanisms of microRNAs in the regulation of cellular processes [4, 7, 68]. However, this area remains largely unexplored.

In addition to the fact that microRNAs located in the cytoplasm execute translational repression, their ability to activate translation when binding non-polyadenylated mRNAs was shown [69, 70]. The miRISC components were also found to be localized in several subcellular compartments, including the rough endoplasmic reticulum [71], processing P-bodies [72], stress granules (SG) [73], trans Golgi network, early/late endosomes, multivesicular bodies (MVB) [74], lysosomes [75], and mitochondria [76, 77]. In 2004, Meister et al. [78] identified the first microRNA (microRNA-21) in the nucleus. About 20% of mature microRNA-21 isolated from the Hela cells were distributed in the nucleus. Hwang et al. [79] reported that human microRNA-29b is predominantly localized in the nucleus of mitotic cells. Thus, both microRNAs and individual components of the RISC complex can freely circulate between the cytoplasm and the nucleus, penetrating into the nucleus and binding to proteins of the karyopherin family, in particular exportin-1 XPO1, importin-8 IPO8, karyopherin p KPNB1 and XPO5, subject to availability of a nuclear localization signal (NLS) and a nuclear export signal (NES). The RISC components such as TNRC6A and DICER contain NLS and translocate from the cytoplasm to the nucleus by binding to IPO8 and KPNB1, respectively [80-82]. AGO2 does not contain a classical NLS, but IPO8 colocalizes with AGO2 in human and mouse cells, with only microRNA-loaded AGO2 translocating into the nucleus. Once in the nucleus, AGO2 is involved in repair of double-strand cuts [83] as well as in regulation of the chromatin remodeling complexes functioning [84, 85]. When it enters the nucleus, AGO1 binds to RNAPII and the promoters of actively transcribed genes and regulates the expression of genes that participate in oncogenic pathways such as cell cycle progression, growth, and survival [86, 87]. AGO1 and AGO2 can regulate alternative splicing by inducing H3K9me3 at variant gene regions, which results in the RNAPII inhibition and spliceosome recruitment [88]. Analysis of the microRNA circulation between the nucleus and the cytoplasm revealed that the nuclear and cytoplasmic fractions of microRNAs are not equally enriched. The duration of microRNA presence in the nucleus correlates with the number of predicted complementary targets in it. Moreover, microRNA effector complexes in the nucleus have a significantly lower molecular weight compared to the cytoplasmic ones [89].

Further, we shall consider some examples of the microRNAs functioning in the nucleus, such as regulation of the histone code, interaction with promoters, enhancers and transcripts. MicroRNAs are powerful epigenetic regulators as they control a large number of transcripts responsible for chromatin rearrangements [90]. MicroRNAs can also regulate the activity of enhancers by means of translational repression of enhancer-binding proteins, as it is shown for C/EBP α, β (transcription factors that are key regulators of glucose and lipid metabolism in the liver), microRNA-21 and microRNA-155 [91, 92]. It is worth noting that microRNAs also directly bind to and regulate gene enhancers (NamiRNAs) to activate transcription [93]. Studies in cell lines [94] identified >300 microRNA loci in genomic regions with active enhancer markers, such as DNAse I hypersensitive sites, histone H3 Lys 27 acetylation modification (H3K27ac), and recruitment of the p300/CBP coactivator complex. A recent study [95] demonstrated the role of microRNA-492 as a trigger of enhancers in pancreatic cancer development.

In the context of liver cell populations, non-canonical functions of microRNAs are still underexplored. They may indirectly participate in chromatin remodeling by means of translational repression of key participants of this process. MicroRNA-885-5p, which is recognized as a marker for HCC progression, is an example of that. Zou et al. [96] revealed an increase in the level of H3K4me3 histone, which is a marker of transcriptional chromatin accessibility. The authors believe that the effect is associated with decondensation of the *TIGAR* gene promoter and increase in its transcription.

Another mechanism of transcription regulation by microRNAs is formation of a complex of microRNA-589-5p with AGO2 and GW182 in the nucleus. The complex directly binds to the promoter of cyclooxygenase-2 (*COX-2*), thus activating its transcription [97] and promoting development of liver fibrosis and the HCC progression [98-100]. MicroRNA-552 regulates the cytochrome P450 (CYP2E1) expression at the transcription and translation levels. MicroRNA-552 interacts with a cruciform structure in the promoter region of the cytochrome, which prevents binding of the transcription factors SMARCE1 and RNA of polymerase II [101].

By binding to the TATA box promoter, microRNA let-7i can activate transcription of the *IL2* gene, making the assembly of the initiation complex easier and attaching the TFIID, TFIIA, TFIIB, TFIIE, and TFIIF transcription factors [102]. Moreover, it was established that the expression of FOXP3, a key transcription factor for formation and development of T-regulatory Treg lymphocytes, is regulated similarly. It was established that IL-2/STAT5 signal transduction increases the FOXP3 expression not only in the classical way, but also by enhancing the expression of the microRNA-4281 precursor gene, which, by binding to the TATA-Box of the *FOXP3* gene, additionally activates its transcription [103]. Kurt et al. [104] demonstrated the role of *IL2*-mediated Treg recruitment to reduce inflammatory damage in the CCl4 model of toxic liver damage.

During the transcription of the host gene, nuclear microRNAs can also form a functional positive feedback loop. For example, microRNA-483-5p, encoded in the *IGF2* gene, can transcriptionally increase its expression, which results in the HCC increased progression *in vivo* [105].

Besides protein-coding genes, nuclear microRNAs also regulate biogenesis of non-coding RNAs at the transcription level. In particular, MALAT-1 is a highly conserved lncRNA, which plays a regulatory role in regenerative processes and liver diseases, including fibrosis, steatosis, and liver cancer [106]. It was reported that microRNA-9 can bind to AGO2 in the nucleus and regulate the MALAT-1 transcription [107]. Moreover, nuclear microRNAs can also interact with other pri-microRNAs and regulate biogenesis of the corresponding microRNAs. For example, microRNA-709 includes 19 nucleotides that are completely complementary to the pri-microRNA-15a/16-1 sequence. MicroRNA-709 suppresses the processing of pre-microRNA-15a/16-1 from pri-microRNA-15a/16-1 and finally reduces the level of mature microRNA- 15a/16-1, which results in cell apoptosis [108].

It was found in the study [109] that microRNA-122 in the nucleus binds to a 19-nucleotide UG- containing recognition element in the basal region of pri-microRNA-21 and prevents the Drosha DGCR8 microprocessor from converting pri-microRNA-21 into pre-microRNA-21. This mechanism is important for cell growth and proliferation because microRNA-21 regulates programmed cell death protein 4 (PDCD4), which is a tumor suppressor. Therefore, this non-classical action of microRNA-122 explains its proapoptotic effect.

## Role of microRNAs in maintaining homeostasis and development of liver pathology

At present, there are several large studies aimed at identification of microRNAs specifically expressed in the liver. These studies consider the diversity of secreted and intracellular microRNAs both at the organ and specific cell levels.

### MicroRNAs regulating liver metabolism

Multiple reviews highlight the role of microRNAs in regulating energy metabolism and detoxification function of the liver, as well as their contribution to development of various pathologies [110-116]. In their recent review, Goncalves et al. [117] assessed the impact of differential expression of microRNAs on metabolism of lipids, carbohydrates and development of insulin resistance, fatty liver disease, as well as the role of the liver-derived microRNAs in development of cardiovascular pathologies.

A large number of studies are focused on the search of microRNA patterns specific to tissues and cell types, as well as on identification of its role in homeostasis and pathology development. A classic example of a tissue- specific microRNA is microRNA-122, which takes over 70% of the liver miRNome [118], but the increased levels of this microRNA are also associated with development of metabolic syndrome, and diabetes in particular, and the overall risk of mortality in patients with heart failure [119, 120]. Despite the fact that microRNA-122 is expressed predominantly by liver cells, overexpression of microRNA-122 in cardiomyocytes was demonstrated; it promotes cardiomyocyte apoptosis and development of multiple cardiac pathologies [121]. Over 120 targets were annotated for microRNA-122 [122], they are associated with a wide range of cellular processes. MicroRNA-122 plays a key role in maintaining liver homeostasis as it modulates the expression of the Cyclin G1, ADAM10, IGF1R, SRF, and Wnt1 proteins [123], and regulates hepatocyte differentiation by targeting components of the Hippo signaling pathway [124].

Besides microRNA-122, transcriptome profiling identified a total of 277 microRNAs expressed in the liver, 166 of which were expressed in all analyzed samples, including microRNA-16, microRNA-27b, microRNA-30d, microRNA-126, and several members of the let-7 family [125]. Various studies also described the role of differentially expressed microRNAs in development of pathologies. For instance, hepatocyte-specific functions were described for microRNA-155 in the context of alcoholic liver disease and a partial hepatectomy model [126, 127], as well as for microRNA-192 in acute liver damage and liver fibrogenesis mediated by HCV infection [128].

MicroRNAs involved in regulation of stellate cells, which produce extracellular matrix of the liver, were identified. Activation of proliferation and synthetic activity of stellate cells is mediated by microRNA-130 through the sirtuin 4 (SIRT4) repression [129], and microRNA-21 by activation of the PTEN/Akt signaling pathway [130]. TGF-β-induced activation of microRNA-199 and microRNA-200 indirectly advances liver fibrosis by increasing the expression of profibrotic genes (e.g., collagens, matrix metalloproteases MMP) [131]. Some microRNAs have an antifibrotic effect by suppressing the stellate cells activation and blocking the expression of the extracellular matrix specific components. For instance, microRNA-29 and microRNA-19b suppress the expression of the transforming growth factor receptor beta II (TGFβRII), thus reducing the stellate cells activation [114, 132]. Similar to microRNA-29, the main regulator of fibrosis [133], members of the microRNA-17-92 cluster (including microRNA-19b [134]) are involved in the control of stellate cell activity and liver regeneration in a great extent [135].

In contrast to the widely explored microRNAs of hepatocytes and stellate cells, data on specific functions of microRNAs in Kupffer cells and cholangiocytes are not numerous. It was suggested that microRNA-155 is activated in infiltrating and resident macrophages (Kupffer cells) under alcohol exposure, which promotes development of inflammation and fibrosis by regulation of the TLR4/NF-ΚB, Keap1/Nrf2 pathways, and this ultimately results in steatosis, hepatitis and cirrhosis [126, 127, 136]. MicroRNA-223, in addition to maintaining lipid homeostasis, regulates activation and polarization of immune cells, which is discussed in detail in the review [137]. MicroRNA-223 promotes acute neutrophil response by targeting IKK-α expression in acetaminophen-induced acute toxic liver damage [138].

In general, microRNAs actively regulate energy metabolism in liver cells. They maintain mitochondrial homeostasis; violation of regulation leads to mitochondrial dysfunction [139]. A complex regulatory network of microRNAs maintains liver homeostasis, ensures response to stress impact, and coordinates various molecular cascades. Below are several examples illustrating the role of microRNAs at various levels of regulation in liver cells. Sirtuins (SIRT1- SIRT7) play key roles in energy/lipid metabolism, oxidative stress, inflammatory response, mitochondrial homeostasis, autophagy, and necroptosis by regulating multiple signal transduction pathways [140-143]. Despite the large number of microRNAs identified as regulators of sirtuins in various tissues, there is little data about their effect on liver sirtuins. SIRT6, like SIRT1, is predominantly localized in the nucleus, where it is involved in regulation of glycolysis and triglyceride synthesis, as well as β-oxidation of fatty acids [143]. It was demonstrated [144, 145] that the microRNA-33a/b group negatively regulates the SIRT6 expression in liver cells. It is of interest that microRNA-122 also has a binding site in the 3’ non-coding region of SIRT6 and mediates its negative regulation in adventitial fibroblasts and liver, where they co-regulate fatty acid oxidation [146, 147].

The main deacetylase in mitochondria, SIRT3, is involved in all aspects of mitochondrial metabolism, as well as in mitochondrial biogenesis and dynamics, protecting against ROS and regulating the tricarboxylic acid cycle. In addition to easing mitochondrial stress, SIRT3 can trigger mitophagy by activating FOXO3a [143]. It was established that microRNA-34a-5p [148, 149] and microRNA-421 (which significantly increases in case of NAFLD [150]) can directly or indirectly regulate SIRT3, thus suppressing its gene expression and protein levels in the liver. Moreover, microRNA-210 targets and suppresses the iron-sulfur cluster assembly enzyme (ISCU), which changes the NAD+/NADH ratio, indirectly affecting SIRT3 [151].

SIRT1 is part of numerous metabolic pathways such as gluconeogenesis, glycolysis, fatty acid oxidation and synthesis, oxidative phosphorylation or nitrogen metabolism, as well as of several fundamental and homeostatic processes such as mitochondrial biogenesis, inflammation, apoptosis, or oncogenesis [142]. SIRT1 is regulated by microRNA-19b [152], microRNA-22 [153], and microRNA 449a [154].

One of the sirtuin functions is deacetylation, which is required for activation of the PGC1a protein being the main controller of mitochondrial biogenesis, which plays a vital role in regulation of cellular energy metabolism [155]. *PGC1α* was identified as a target gene for microRNA-871-5p [156], microRNA-29c [157] in hepatocytes. It was demonstrated that NAFLD is characterized by a decrease in the levels of mRNA and the PGC1α protein, as well as by impaired binding to the promoters of the nuclear respiratory factors NRF1 and NRF2 [158], which define the expression of many nuclear genes that encode proteins targeting mitochondria, such as DNA polymerase y (POLG), DNA helicase (Twinkle). These proteins are required for mtDNA replication and mitochondrial transcription factor A (TFAM) [159]. For instance, overexpression of microRNA-378a-3p suppressed *NRF1*, promoting accumulation of lipids and violation of fatty acid oxidation, which resulted in hepatosteatosis aggravation [160, 161].

Guo et al. [162] demonstrated the role of microRNA-199-5p in regulation of glycolysis in the HCC cells. Suppression of microRNA-199-5p expression by hypoxia-induced factor-1α promoted tumor progression within the Warburg effect. The direct target of microRNA-199-5p, microRNA-885-5p [163], and microRNA-125b [164] in hepatocytes is hexokinase 2 (HK2), which catalyzes the first irreversible stage of glycolysis. A shift in energy metabolism towards glycolysis is also influenced by an increase in pyruvate dehydrogenase kinase 4 (PDK4) [165, 166], which in turn makes the tumor more aggressive, but reduces the efficiency of liver regeneration in a partial hepatectomy model [167, 168]. The regulation of PDK4 in the liver is mediated by microRNA-9-5p [169] and microRNA-129-5p [170], and microRNA-155 regulates PDK4 by the C/EBPβ pathway [171].

Change of the redox state is an important basis for many liver diseases. The redox state varies with the progression of inflammatory, metabolic, and proliferative liver diseases. In the mitochondria and endoplasmic reticulum of hepatocytes, enzymes of the cytochrome P450 family participate in the reactive oxygen species (ROS) production. Under appropriate conditions, cells initiate specific molecular cascades that control the level of oxidative stress and maintain the balance between oxidative and antioxidant components [172]. The redox-sensitive transcription factor Nrf2 is a cellular redox sensor, which, with the ROS levels increase, promotes the transcription of genes that protect cells and tissues from oxidative stress. Genes regulated by NRF2 by means of antioxidant response elements (AREs) include ROS-related factors involved in glutathione metabolism, reduction of the oxidized protein thiol groups, and NADP-producing enzymes, which are required for medicine-metabolizing enzymes and antioxidant systems [173]. In the context of liver pathology, NRF2-mediated cytoprotective responses prevent the development of various diseases of this organ, including alcoholic and non-alcoholic liver diseases, viral hepatitis, fibrosis, and HCC. The NRF2 activity is regulated in the liver by several microRNAs, in particular, microRNA-27a, microRNA-142-5p, microRNA-153, and microRNA128 [174]. Regulation of the NRF2 stability is performed by microRNA-200a and microRNA-125b-5p, targeting KEAP1, which facilitates the NRF2 degradation and increased oxidative distress in fibrosis and lipid disorders [175, 176]. Moreover, microRNAs are actively involved in regulation of other components of the response to oxidative stress in the liver [177, 178].

Similar to oxidative stress, endoplasmic reticulum stress (ER stress) is an important part of the liver diseases pathogenesis [179-181]. Moreover, these conditions are closely linked [182-184]. For instance, NRF2, which is a key participant in the response to oxidative stress, may contribute to protection from ER stress by activating SIRT3 [185]. Lipid metabolism violation leads to oxidative and ER stress. ER stress triggers activation of three transmembrane ER sensors: IRE1α/β, PERK, and ATF6, which facilitate cell adaptation to stress [186].

Activated IRE1α results in degradation of microRNAs, such as microRNA-17, microRNA-34a, microRNA-96a, and microRNA-125b, which are related to cell protection under stress conditions, by means of post-transcriptional degradation of the apoptotic cell death regulator caspase-2, which is a thioredoxin- interacting protein TXNIP, etc. [187-189]. The most studied of these microRNAs is microRNA-34a, the cleavage of which results in steatosis relief by means of activation of β-oxidation, fatty acid transport, and apoptosis (provided in detail in [190]). Activation of PERK induces the expression of microRNA-211, which in turn targets the chop/gadd153 promoter and attenuates its expression. This action allows the cell to restore homeostasis before triggering apoptosis [191]. MicroRNA-211 also suppresses the translation of circadian regulator Bmal1, which contributes to inhibition of the protein synthesis, which is required to restore cellular homeostasis [192]. It is currently believed that the IRE1 and PERK signaling branches are opposed and provide for cell fate determination in line with the severity of ER stress [193, 194]. The existing antagonism is not only due to the protein-protein interaction. It is assumed that microRNA-30c-2, activated by PERK in combination with the inflammatory transcription factor NF-KB, targets the main effector of the IRE1 branch, the XBP1 transcription factor, regulates its turnover, and maintains the balance between pro-adaptive and non-adaptive processes in ER stress [195]. The ER stress relief in hepatocytes may be facilitated by microRNA-26a [196] by targeting the eukaryotic initiation factor eIF2α, which is also inhibited with activation of PERK. It should be noted that the expression of microRNA-26a is suppressed in models of non-alcoholic fatty liver disease and in the liver of patients with NAFLD. A recent study established that ATF6 is a direct target of microRNA-149 and may relieve ER stress in case of NAFLD by creasing inflammatory response and preventing caspase 12 activation [197].

Therefore, microRNAs ensure regulation of a wide range of metabolic processes in various cell populations of the liver. Change of their expression has an adaptive function and is also part of the pathology progression mechanism.

### Intercellular communication

Modern researchers consider extracellular vesicles as a key mediator of intercellular communication, as they conduct both local autocrine, paracrine, and endocrine regulation. Various aspects of biogenesis and functioning of extracellular vesicles under normal conditions and in various pathologies are being actively studied. Reviews [198-200] provide the current views on the biology of extracellular vesiclesin details.

There is no doubt now that molecules located in extracellular vesicles reflect pathological processes in cells [201-204]. Since the discovery of the possibility to transport mRNA and microRNA in vesicles in 2007 [205], the regulatory role of nucleic acids has been most actively studied. In recent years, several databases and community-generated catalogs of molecules identified in extracellular vesicles were created, such as Vesiclepedia (Kalra et al. [206]), ExoCarta (Mathivanan and Simpson [207]), exRNA (Murillo et al. [208]), and exoRBase (Lai et al. [209]).

The study [210] identified that 210 out of 664 (34%) analyzed microRNAs are differentially expressed in extracellular vesicles from different types of cells, which corresponds to the views on specific microRNA profiles and mechanisms of their distribution (sorting) typical for different cells. The authors also demonstrated that (depending on the cell type) certain microRNAs can be either preferentially exported or, vice versa, kept in the cell. Santangelo et al. [211] identified a microRNA sorting system in hepatocytes. The SYNCRIP protein mediates the transport of microRNAs with the hEXO (GGCU) motif into vesicles; GGCU was common to approximately 60% of microRNAs, predominantly found in exosomes. The authors believe that this mechanism can work both independently and synergistically with other mechanisms, for example, with the previously identified hnRNPA2B1-GGAG [212].

For some microRNAs, intracellular changes corresponded to the observed changes in microRNA concentrations in serum. Activation of microRNA-571 in hepatocytes and stellate cells was accompanied by increased levels of this microRNA in the blood serum, which correlated with the liver fibrosis stage in patients, whereas lower levels of microRNA-652 in serum correlated with the decreased expression of this microRNA in monocytes and reflected the degree of the liver inflammation [213].

Asymmetrical sorting of microRNA-122 in the liver in case of NAFLD was also reported. MicroRNA-122 is a key regulator of lipid homeostasis in the liver [214], and its increase was identified in case of NAFLD development [215, 216]. The authors [217] found that in NAFLD there is an increase in the level of extracellular microRNA-122, but the level of the same microRNA in hepatocytes abruptly decreases. This may be a part of the regulatory mechanism described by O’Grady et al. [218]. According to their results, the HNRNPA2B1 protein can mediate the regulation of the cellular transcriptome by getting involved in packaging of microRNAs, mRNAs, and lncRNAs into vesicles, which allows the cell to avoid unwanted transcripts.

Besides extracellular vesicles, the microRNAs transfer is conducted by albumins [219], high-density lipoproteins (HDL) 5 to 12 nm in size, low-density lipoproteins (LDL) 18 to 25 nm in size, chylomicrons up to 1200 nm in size, which are wide-spread in blood circulation and probably are the predominant type of particles in plasma preparations [220-223]. HDL and LDL, like chylomicrons, can also transport microRNAs, and thus are important transporters of microRNAs in the blood flow [224, 225]. Considering the fact that the liver is one of the main sources of lipoproteins in the blood, one can conclude that the diagnostic potential of microRNAs transported by lipoproteins is enormous. However, the difficulty of conducting experiments on isolation and studying lipoprotein fraction separate from extracellular vesicles makes these studies extremely problematic.

Wagner et al. [226] analyzed microRNAs transported by blood lipoproteins. The results of the study show that HDL is richer in microRNAs compared with LDL. Here, the authors suggest that 8% (over 10,000 copies/pg) of circulating microRNA-223 are associated with HDL.

The first proof of biological functionality of microRNAs transported by HDL was presented in 2011 by Vickers et al. [227]. The authors showed that native HDL loaded with microRNA-375 or microRNA-223-3p mimics efficiently delivered these microRNAs into the cultured human hepatocytes with a decrease in the mRNA levels of two putative target genes of microRNA-223-3p in the background. They found that the transfer of lipoprotein-associated microRNA into the recipient cells was primarily dependent on the scavenger receptor B type 1 (SR-BI).

### MicroRNAs and viral diseases

In case of viral infection, microRNAs act as regulators of viral replication, antigen presentation, and immune response. They can also cause a wide range of cytotoxic effects. Viruses not only cause aberrant expression of host microRNAs, but also encode their own microRNAs [228]. Encoding of microRNAs by viruses was first reported in 2004, when the first microRNAs of the Epstein-Barr virus were found [229]. Currently, 44 mature microRNAs are known to be encoded in the Epstein-Barr virus genome [230, 231]. Recent studies demonstrated availability of the viral microRNA CoV2-miR-O7a in coronavirus. Researchers assume that this microRNA regulates interferon signal transfer [232-234].

According to the WHO estimates, in 2015, 257 million people (3.5% of the population) lived with the chronic hepatitis B (HBV) infection; 2.7 million of them were coinfected with HIV (https://www.who.int/publications/i/item/9789241565455).

Infection with HBV not only causes hepatitis, but also increases the risk of HCC by 100-fold [234]. However, the currently available data do not allow scientists to comprehensively describe the processes underlying malignant transformation.

The first microRNA of the HBV virus was identified quite recently, in 2017. HBV-miR-3 is located at nucleotides 373 to 393 of the HBV genome [235]. HBV-miR-3 targets a unique region of the HBV transcript with 3.5 thousand pairs. Later, the authors [236] found that this microRNA can enhance the interferon-mediated antiviral response, as well as promote the M1 macrophages polarization and enhance the secretion of IL-6 by means of direct inhibition of SOCS5. HBV-miR-3 binds to the 3’ region of the tumor suppressor PTEN mRNA, suppressing its translation, which facilitates tumor cells avoiding apoptosis and their increased proliferation [237]. Another microRNA encoded by the hepatitis B virus was discovered in 2022 by Loukachov et al. [238]. HBV-miR-6 is located between nucleotides 255 and 325 of the HBV genome. The authors assume that it is involved in replication and release of viral particles, as the mRNA level of none of the 25 potential targets identified by miRDB was changed by overexpression of HBV-miR-6, whereas its expression level correlated with the level of HBV DNA in the liver and the HBsAg surface antigen in plasma. Unlike the previously identified in hepatoma samples HBV-miR-3, HBV-miR-6 was found at earlier stages of disease development, which may indicate a switch in microRNA expression at different stages of disease.

Cellular microRNAs can also influence replication of viral genomes and regulate antiviral response. MicroRNA-122 is an example of such microRNA, it promotes replication of the HCV by protecting it from degradation and changing the viral RNA conformation, making the initiation translation site (IRES) accessible to enzymes [239-242].

## MicroRNAs in clinical practice: diagnostic and therapeutic capacities

### MicroRNA-based diagnostics

Due to significant scope of accumulated knowledge about biological function of microRNAs and changes in microRNA during the development of various pathologies, microRNAs are currently being actively studied as a diagnostic, prognostic, and therapeutic tool for clinical practice. There are currently 509 results for the “microRNA” query in the ClinicalTrials.gov clinical trial database (https://clinicaltrials.gov), but only 35 of them contain liver-related key terms, though the vast majority of studies focus on liver-related diagnostics based on microRNAs. In their review, Kim and Croce [243] consider promising clinical trials of diagnostic panels and microRNA-based medicines for cancer treatment. The authors emphasize that the pleiotropy of microRNA action is both an obstacle to development of microRNA therapy due to unexpected off-target effects, and a key advantage as carcinogenesis is characterized by complex underlying epigenetic changes. Liver diseases are no exception to this. In a recent review, Zhao et al. [244] thoroughly reviewed biological function, participation in pathogenesis, and diagnostic potential of microRNAs of extracellular vesicles secreted by different types of liver cells.

MicroRNAs can act as new biomarkers for various pathologies because they are very stable and easy to detect in peripheral blood. Studies on gene expression profiling identified changes in microRNA expression in a number of human diseases. The complexity of microRNA regulation does not allow to use only one microRNA as a marker of a particular disease. Huge efforts are invested into finding panels of microRNAs that could help diagnose a disease with high accuracy, track the disease progression, and adjust the treatment regimen accordingly. Multiple microRNA panels are being actively developed to diagnose HCC [245, 246], fibrosis [244, 247], steatosis [190, 248], acute liver transplant rejection [249], and other pathologies.

Experiments to identify microRNA biomarkers typically include a discovery phase and a validation phase. During the discovery phase, one hundred microRNAs are analyzed in parallel to identify candidate biomarkers. Due to high cost of high-throughput experiments, the number of people involved in such studies is often too small, which can easily result in false positive and false negative findings. During the validation phase, a small number of identified biomarker candidates are measured in a large sample of test and control samples, typically using quantitative PCR (qPCR). Although qPCR is a sensitive method to measure microRNAs in blood, the design of experiment and analysis of qPCR data remain a weak point in many studies. Omitting important stages when planning and analyzing qPCR in the experiment or inappropriately performing thereof can lead to serious systematic errors. Detailed recommendations related to planning and conducting experiments to identify RNA and microRNA biomarkers are provided in [250-252].

### MicroRNA-based therapeutic approaches to treatment of liver diseases

**Figure 2.**
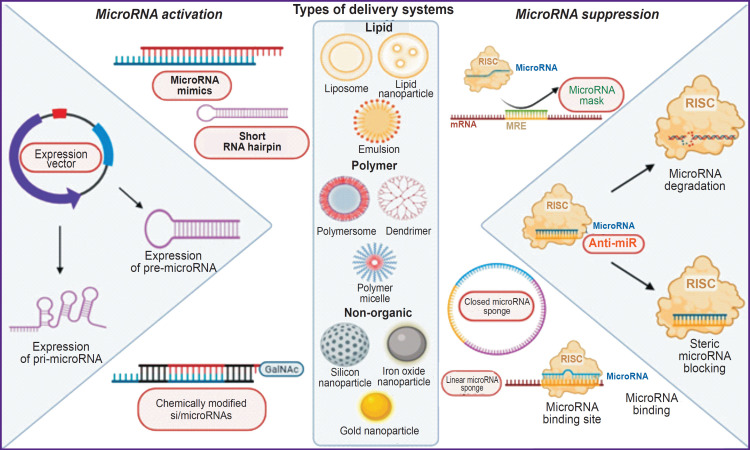
Nucleic acid delivery ways and main microRNA-based therapeutic strategies

Manipulation with the microRNA expression level can simultaneously influence a wide range of clinically important targets, thus opening promising therapeutic prospects. Activities on development of microRNA-based therapy can be divided into two large groups: microRNA replacement therapy (enhancement or restoration of the expression of endogenous microRNAs acting as suppressors of pathology) and microRNA suppression (inhibition of the expression or functional blocking of microRNAs acting as drivers of pathology). Figure 2 demonstrates methods of nucleic acid delivery and main therapeutic strategies on the basis of microRNAs.

Change of the level of microRNA expression is usually conducted with the use of nucleic acids, including oligonucleotide-based microRNA inhibitors (anti-miRs), microRNA sponges, etc., as well as microRNA agonists: synthetic microRNAs (miRNA mimics), recombinant expression vectors that carry sequences encoding microRNAs, etc. One shall specifically emphasize therapeutic approaches based on the use of bioactive substances and ligands that affect the transcription, processing and functioning of microRNAs [253]. In their review article, Doghish et al. [254] provide examples of microRNAs considered as therapeutic targets for inhibitors or replacement therapy for various liver diseases. In their meta-analysis, Zhu et al. [255] mentioned 96 studies that examined therapeutic effects of 56 various microRNAs on NAFLD/NASH. The authors note the role of microRNA-34a and microRNA-21, as well as the microRNA-130 and microRNA-146 families in development of liver pathologies.

Decrease of microRNA activity is mainly achieved by using microRNA sponges, antisense oligonucleotides (ASOs) masking microRNAs, or antisense oligonucleotides targeting microRNAs (AMOs) [256]. MicroRNA sponge technique allows triggering the expression of mRNA molecules with multiple binding sites for the target microRNA, which further act as a recall or sponge to capture targeted microRNA. Thereby, the endogenous target mRNA is preserved and capable of normal functioning [257]. Like endogenous microRNA sponges, the introduced structures can be linearized and circularly closed (like endogenous circular RNAs), which increases their stability and reduces off-target effects [258, 259]. A significant limitation of this technique is its high cost. Often, viral vectors are also must be used for their delivery into the cell, as well as selection of promoters that ensure high expression in a specific cell type. Moreover, such structures are characterized by high immunogenicity and cytotoxicity. However, microRNA sponges can carry numerous microRNA binding sites, thus allowing simultaneous regulation of a large number of targets.

Antisense oligonucleotide technique that masks microRNA targets (miR-mask, also known as the BlockmiR, target protectors or target site blockers) is based on the reverse approach: instead of blocking a target microRNA, these molecules protect the mRNA, functioning of which one wants to keep. Moreover, miR-mask technique targets microRNAs in a gene-specific manner, meaning that oligonucleotides are designed to protect specific sites of the mRNA and, correspondingly, the expression of the considered protein. This allows to keep other transcripts regulated by microRNAs unaffected, which helps reduce off-target effects of therapy [260]. One of potential obstacles to the use of miR-mask may be availability of multiple binding sites for the microRNA seed region in the encoding and 5’ regions of mRNA.

This approach can be further combined with microRNA sponges (Sponge miR-mask technique) to block access of several microRNA members to their binding sites on the mRNA, which results in activation of protein expression. Sponge miR-mask is designed to bind by means of partial complementarity to the 3’UTR of all target germ site mRNAs of the microRNA family of 8 nucleotides. However, Sponge miR-mask technique has poor gene specificity, as these molecules can block the expression of all genes associated with the same target binding site of the whole microRNA family [261].

The most popular approach to correct aberrant microRNA expression is based on synthesis of antisense oligonucleotides with a microRNA-complementary sequence. To improve biostability and affinity of binding to the target microRNA, anti-miRs need to be chemically modified. Modifications include introduction of phosphorothioate bonds into internucleotide bonds, modification of the 2’-O-methyl RNA sugar (2’OMe) in antagomiRs, or introduction of locked nucleic acid (LNA) bases in LNA-anti-miR. Anti-miRs with lower affinity chemical modifications, like 2’OMe, induce microRNA degradation, whereas anti-miRs with chemical modifications increasing target affinity, such as LNA, do not induce microRNA degradation but inhibit the target microRNA using a steric blocking mechanism [262]. While antagomiRs are conjugated to cholesterol, which facilitates their cellular uptake, LNA-anti-miRs have a phosphorothioate backbone, thus ensuring greater stability, high binding affinity, and good pharmacokinetic properties [263]. Miravirsen (anti-microRNA-122) is an antisense LNA oligonucleotide to microRNA, which was a first to be used in clinical trials as a targeted medicine for the HCV treatment [264].

MicroRNA replacement therapy is aimed at restoration of the microRNAs level, lowering of which contributes to the pathology development. Synthetic double-stranded microRNAs (miRmimic) carrying various chemical modifications, some of which are covered below, can be used as exogenous microRNAs. MicroRNAs can be delivered into the cell in the form of a nuclear transcribed vector, which then goes a classical pathway of microRNA biogenesis from the pri-microRNA stage [265] or pre-microRNA stage [266]. Moreover, small interfering RNAs (siRNAs), acting like microRNAs with high complementarity to the target, have a scarcer repertoire of targets compared to canonically paired microRNAs due to complementary regions outside the seed region, which also allows AGO2 to cleave mRNA within endonuclease activity, providing a knockdown efficiency of over 80% [267]. Despite the simplicity of design and a wide range of targets of microRNA mimics, siRNAs have fewer off-target effects, which is their advantage in clinical trials.

It should be noted that double-stranded microRNA mimics can potentially induce a nonspecific interferon response through TLR-dependent and TLR-independent pathways [268]. Another potential problem of microRNA replacement therapy is the problem of administering supraphysiological concentrations of microRNA trying to restore microRNA levels, which leads to uptake into non-target tissues, as well as inclusion of passenger strands in RISC, and accumulation of microRNA modification products. Therefore, targeted delivery of microRNA mimics to the appropriate cell or tissue type is important to prevent adverse side effects of this therapeutic approach.

### Delivery systems

Bioactive molecules acting as therapeutic agents are delivered by means of passive and targeted delivery. Passive delivery is determined by its intrinsic properties and anatomy of the tissue or cell type. Specific ligands or recognition molecules for passive medicine delivery are not required [269]. A function of the mononuclear phagocytic system (MPS) is to capture and remove foreign bodies from the circulatory system to protect the body from harmful effects. Consequently, microRNAs, provided they are stable enough, tend to accumulate through passive delivery in the liver, spleen, lymph nodes, and kidneys, which are filtering organs of the MPS [270].

Unlike most other tissues, the liver does not have an impermeable basement membrane. Therefore, in the absence of obstacles such as aggregation or protein binding [271], most microRNA carriers exhibit fast passive accumulation in the liver after systemic administration. Passive targeting to liver cells is generally determined by the diameter of the fenestrae formed by liver sinusoidal cells, which provide selectivity for Kupffer cells and sinusoidal cells (>100 nm) on the one hand, or hepatocytes and stellate cells (<100 nm) on the other hand. In tumor tissues, the nanoparticles accumulation can be significantly eased due to the typical permeable tumor vasculature and the increased distance between vascular endothelial cells [272].

Despite the effectiveness of passive uptake of nanoparticles by the liver, many off-target effects resulting from accumulation in the kidneys, lungs, spleen, and other organs create the need for a more specific targeted delivery. There are several approaches to active targeted delivery of microRNAs that include conjugation, virus-associated delivery, and modified nanoparticles. Though there is experimental evidence that virus-associated microRNA delivery approaches are effective in cancer treatment, safety concerns regarding the use of viruses currently limit their clinical application, and other non-viral delivery systems are considered more promising [273].

The conjugation method, in which lipids or ligands targeting cellular receptors directly bind to microRNAs, is one of the popular approaches to microRNA delivery. The liver actively uptakes a wide range of high and low molecular weight compounds, which allows to highly efficiently use microRNAs connected to various ligands. One can have asialoglycoprotein receptor 1 (ASPGR1) as a specific targeting site in the liver; this receptor is a transmembrane protein expressed predominantly on the hepatocyte membrane [274]. Specific binding of N-acetylgalactosamine (GalNAc) to ASPGR1 leads to rapid endocytosis. As of August 2023, four GalNAc-conjugated siRNAs (givosiran, lumasiran, inclisiran, and vutrisiran) produced by the Alnylam Pharmaceuticals biopharmaceutical company were approved for clinical application. Givosiran is designed to inhibit hepatic 5-aminolevulinic acid synthase 1 (ALAS1) to treat acute hepatic porphyria caused by disruption of ALAS1 expression, which can result in accumulation of toxic metabolites. Lumasiran is used to treat primary hyperoxaluria type 1 by inhibiting the expression of hydroxy acid oxidase 1 (HAO1), which results in the decreased oxalate levels in the liver. Inclisiran reduces the level of expression of hepatic protein convertase subtilisin/kexin type 9 (PCSK9), which results in a decrease in the LDL cholesterol levels. This medicine is used to treat hypercholesterolemia, characterized by the increased levels of LDL cholesterol, which is associated with cardiovascular risks. Vutrisiran targets transthyretin (TTR) mRNA and reduces the levels of TTR protein in blood; this protein is primarily produced by the liver. The medicine is used to treat amyloid-transthyretin-mediated (ATTR) amyloidosis. Willoughby et al. [275] demonstrated that in case of 50% decrease in ASPGR expression, the efficiency of GalNAc-siRNAs conjugates uptake is maintained; it makes one assume that there are independent mechanisms of internalization available and this medicine may be used to treat pathologies accompanied by a decrease in ASPGR in the liver, including congestive heart failure, alcoholic liver cirrhosis, Laënnec’s cirrhosis of the liver, biliary cirrhosis, as well as liver neoplasms and HCC. GalNAc may also be additionally linked to other functional groups. For instance, Arrowhead Pharmaceuticals used a combination of GalNAc and carboxydimethylmaleic anhydride to create a “proton pump” and release siRNAs from endosomes, thus protecting them from degradation and promoting their release into the cytoplasm [276]. Moreover, cholesterol, lipids, vitamin E (α-tocopherol), and other substances that mediate preferential uptake by a certain type of liver cells which are used as conjugates for microRNA delivery to the liver (the list of substances is given in reviews [272, 277]).

Non-viral carriers for microRNA delivery include liposomes, micelles, dendrimers, and the most commonly used lipid and polymer nanoparticles. Nanoparticles usually include a cationic component that forms complexes with anionic microRNAs, thus protecting them from degradation and allowing interaction with cell membranes to provide for cellular uptake. Lipid-based nanoparticles are the most common class of nanomedicines approved by the FDA or EMA regulators. These carriers have many advantages, such as high biocompatibility, ease of production, high encapsulation efficiency, and flexibility of use, which are important for their clinical application. Liposomes were first introduced into clinical practice in the 1990s as containers loaded with doxorubicin (Doxil) and had a great success in cancer treatment [277]. Then, the most stable solid lipid nanoparticles and nanostructured lipid carriers were developed [278]. Cationic lipids with hydrophilic heads and hydrophobic tails form a complex with the anionic nucleic acid, developing a lipoplex. Cationic lipoplexes may include auxiliary lipids that facilitate targeting to a specific cell type. For example, galactose-modified aromatic lipids are used to target hepatocytes [279]. Moreover, lipoplexes are non-immunogenic and are available as many commercial products, such as Lipofectamine RNAi-MAX, SiPORT (Invitrogen, USA), SilentFect™ (Bio-Rad, USA), and DharmaFECT (Dharmacon, USA). Interaction between the cationic lipids of the lipoplex and the anionic lipids of the endosomes provokes active lipid exchange and development of a gap, which nucleic acids use to enter the cytoplasm [280]. Unmodified cationic lipoplexes were used for microRNA delivery *in vivo*, but their efficiency was low [281]. Several modifications have been used to bypass this problem. Conjugation of the polyethylene glycol (PEG) functional group to cationic lipids helps avoid phagocytosis and agglutination with red blood cells, thus improving the overall efficiency of delivery to the liver [282, 283]. Furthermore, lipoplexes, like other nanoparticles, can be used for co-delivery of medicines. For instance, Xu et al. [284] used DOTAP lipoplexes for co-delivery of doxorubicin and microRNA-101 into the HCC cells. The main disadvantage of cationic lipoplexes is their nonspecific interaction with other proteins, which results in side effects and instability. This problem has been solved by using neutral lipoplexes to deliver microRNAs.

siRNA medicines are being developed based on lipid nanoparticles to fight fibrosis. ND-L02-s0201 contains a siRNA targeting heat shock protein 47 (HSP47), which is required for appropriate folding of procollagen in the endoplasmic reticulum. Lipid nanoparticles include a retinoid-conjugated targeting agent (di-retinamide-PEG-di-retinamide), which promotes the uptake of nanoparticles by target cells (liver stellate cells in liver fibrosis or lung myofibroblasts in pulmonary fibrosis) [285].

Polymer delivery methods often include using polyethylenimines (PEI), in which positively charged aminogroups form a complex with an anionic nucleic acid, thus protecting the RNA from degradation and allowing cellular uptake. Both linear and branched PEIs with low and high molecular weight are used as microRNA carrier systems [286]. However, low transfection efficiency and cytotoxicity make PEI unsuitable for clinical application. To overcome the said limitations, the authors of study [287] used the PEI fluoridation, which reduced its cytotoxicity and led to a more efficient accumulation of nanoparticles in the liver with less off-target accumulation in the lungs. Other polymers, such as polyethylene glycol (PEG) or poly-L-lysine (PLL), help improve its biocompatibility if these polymers are covalently merged with PEI, thus making it less toxic to cells [288].

A copolymer of polylactic acid (PLA) and polyglycolic acid, namely polylactide-co-glycolide (PGLA), is another biodegradable polyester approved by the FDA, which is used for delivery of anti-microRNA [289]. A study [290] showed that PGLA nanoparticles with a size of about 270 nm are predominantly taken up by Kupffer cells. The PGLA hydrophobicity reduces the efficiency of its microRNA delivery. Dendrimers are highly ordered branched polymers that form a complex with nucleic acids based on ionic interactions. Positively charged synthetic polyadenoamine (PAMAM) dendrimers are biodegradable, characterized by higher transfection efficiency and lower cytotoxicity compared with other polymers. Wang et al. [291] in their study managed to successfully deliver intravenous injection of PAMAM and PEG dendrimers, a nanographene oxide bound to anti-microRNA-21, to target tumor tissues. PAMAM dendrimers were used to deliver short activating RNAs (saRNAs) to increase endogenous albumin production with a simultaneous reduction of the tumor burden in the liver [292]. Another approach is to use polymer micelles consisting of a hydrophilic and a hydrophobic polymer. For instance, doxorubicin and the tumor suppressor microRNA-34a were co-delivered into cancer cells within this strategy [293].

### Delivery systems based on inorganic compounds

Inorganic compounds that are used to develop microRNA carriers primarily include gold, Fe_3_O_4_-based magnetic nanoparticles, and silica-based nanoparticles. These thiol- or aminogroup-functionalized nanoparticles can ensure stronger interaction with microRNA, thus easing its delivery [294]. In their article, Li et al. [287] considered the effect of modification of the gold nanoparticles surface with chitosan, PEG and PEI for entrapping by various cells and releasing of nanoparticles into the Disse space of the liver. Silica nanoparticles are thermostable, biocompatible, and have a large surface area and pore size, making them suitable carriers for microRNAs and anti-microRNAs [295]. The advantage of magnetic nanoparticles is their targeted application using a magnetic field. Various modifications of magnetic nanoparticles can prevent nanoparticles aggregation in the magnetic field, as well as reduce their cytotoxicity. A nanocomplex consisting of Fe_3_O_4_ nanoparticles and polymers, namely polyglutamic acid and PEI, demonstrating promising results in the *in vivo* delivery. In xenograft patients, systematic administration of this nanocomplex in combination with the routine chemotherapy with docetaxel suppressed tumor growth, thus improving its therapeutic potential [296]. Magnetic nanoparticles without coating modifications, as well as those coated with dextran, rutin, and methoxy-PEG phosphate, showed predominant accumulation in the liver and spleen [297].

Studies aimed at introduction of microRNAs into clinical practice are subject to general requirements to experiments, however, the current specifics introduce additional control stages that are required to maintain the experiment transparency. General principles of experiments and possible methods to identify target transcripts for small non-coding RNAs are provided in the review by Thomson et al. [298]. The design of the experiment with the use of antisense nucleotides and double-stranded RNAs is described in detail in the recommendations by Gagnon and Corey [299]. The recommendations are not exhaustive and are supplemented by various control stages depending on the specifics of the experiment. Nevertheless, key stages of microRNA therapy development include confirmation of the list of regulated transcripts under conditions, which are as close as possible to the required ones, identification of the cell physiological response to overexpression/knockout of the molecule under study, as well as confirmation of the effect not only by measuring the level of transcription, but also by conducting immunoprecipitation assays to detect changes in the levels of proteins under study. In case of using carriers of a different nature, one shall analyze the cellular uptake, the kinetics of molecules release from the carrier [300], as well as the cytotoxicity of the loaded carrier, as the interferon response can be triggered not only by the carrier, but also by the double-stranded RNA, as it was mentioned above.

## Conclusion

At present, a large amount of knowledge about changes in the microRNA expression in various pathologies has already been accumulated, which allows to create diagnostic microRNA-based panels, but there are many unresolved issues towards the therapeutic use of microRNAs. Developing high-throughput analysis methods help to analyze interactions between various microRNAs and their targets within a cell, assess changes in the proteome, and create models of such interactions for further practical application. Critical role of microRNAs in regulation of cellular processes is out of question; moreover, a major part of the microRNA effects remains unexplored, as they do not fit within the canonical model of microRNA-mediated translational repression. Recent advances in development of small interfering RNA-based therapies to treat liver diseases provide great perspectives for the microRNAs use in treatment of more complex liver pathologies by means of a wider range of regulated targets.
